# Physicochemical Properties, Antioxidant Activities, and Aromatic Profile of Yogurt Co-Fermented by *Weissella cibaria* G232 with Traditional Starters

**DOI:** 10.3390/foods14091607

**Published:** 2025-05-01

**Authors:** Qian Huang, Haixiao Ye, Yangyang Yang, Chenglin Zhu, Junni Tang

**Affiliations:** College of Pharmacy and Food, Southwest Minzu University, Chengdu 610225, China; huangqian0221@163.com (Q.H.); yehaixiao@stu.swun.edu.cn (H.Y.); yang124856@163.com (Y.Y.)

**Keywords:** *Weissella cibaria*, co-fermentation, yogurt, antioxidant, aromatic profile

## Abstract

To improve the quality and functional properties of yogurts, a multi-starters co-fermentation system was used during yogurt preparation. In this work, *Weissella cibaria* G232 (added at 0%, 3%, 5%, and 7%) was involved as a co-fermenter with a traditional starter (*Lactobacillus delbrueckii* subsp. *bulgaricus* G119 and *Streptococcus thermophilus* Q019). The results showed that *W. cibaria* G232 co-fermentation could shorten the fermentation time and significantly enhance the viable counts of yogurt (*p* < 0.05). Moreover, the incorporation of *W. cibaria* G232 improved the water holding ability, viscosity, and texture of yogurt. Notably, the highest levels of firmness, consistency, and cohesiveness of yogurt were observed at the 5% addition level of *W. cibaria* G232. Furthermore, co-fermentation with *W. cibaria* G232 significantly enhanced the antioxidant activity of yogurt, as evidenced by increased free radical scavenging capacity and ferric ion reducing antioxidant power (FRAP) value. The intelligent sensory technology and Gas Chromatography-Ion Mobility Spectrometry (GC-IMS) indicated that co-fermentation with *W. cibaria* G232 and a traditional starter notably altered the accumulation of aldehydes, ketones, and alcohols in yogurt. These findings suggest that co-fermentation of *W. cibaria* G232 with a traditional starter present the potential for the quality and functionality improvement of yogurt and also lay the foundation for the application of *W. cibaria* G232.

## 1. Introduction

Yogurt is a semi-solid fermented product produced by fermenting cow’s milk with a starter culture consisting of *Streptococcus thermophilus* and *Lactobacillus delbrueckii* subsp. *bulgaricus* [1]. Yogurt, known for its distinctive flavor and nutritional benefits, offers multiple health advantages, including maintaining the balance of intestinal microbiota, improving the intestinal barrier, scavenging free radicals, and increasing the body’s resistance to disease [2]. Other benefits are also associated with the consumption of yogurt, such as stimulating peristaltic movements [3], promoting the colonization of the gastrointestinal tract by beneficial microorganisms [4], improving the digestibility of nutrients, and relieving irritable bowel syndrome [5]. However, the quality of plain yogurt could alter during fermentation, storage, and transportation, mainly due to whey separation, low bacterial viability, poor viscosity, and rough texture, which negatively impact the properties of the yogurt [4]. Starter cultures are the key ingredients for the production of yogurt. To address the aforementioned issues, researchers have used probiotic co-fermentation with traditional starters to improve yogurt quality. For example, fermentation by *Lactiplantibacillus plantarum* MTCC 25432 and 25433 could enhance the rheological, texture, and antimicrobial properties of yogurt [6]. *Lactiplantibacillus plantarum* ZFM55 fermentation also distributed a desirable flavor profile of yogurt; at the same time, the water holding capacity, cohesiveness, and viscosity of yogurt improved [7]. *Lactobacillus fermentum* HY01, as an adjunct starter, increased the abundance of volatile flavor compounds in yak yogurt, and 36 volatile substances, including aldehydes, esters, and alcohols, were detected by GC-MS [8]. Although *Lactiplantibacillus plantarum* has been extensively utilized as an adjunct starter culture in yogurt production, persistent acid production during refrigerated storage and mismatched acidification rates with the primary starter cultures may compromise the acid stability and textural properties of the final product. The development of compatible synergistic starter cultures is urgently needed to overcome key challenges in dairy fermentation.

*Weissella* spp. are facultatively anaerobic, Gram-positive, chemoorganotrophs bacteria that do not form spores or produce lactic acid via the hexose-monophosphate and phosphoketolase pathways [9]. The genus *Weissella* belongs to the family *Leuconostocaceae*, and has been frequently found in human and animal intestines, saliva, and traditional fermented foods, such as pickles, sourdough, and cheese [10,11], suggesting it is a lactic acid bacterial genus with Generally Recognized as Safe status [12]. As natural emulsifying stabilizers in yogurt fermentation, exopolysaccharides (EPS) produced by *Weissella* spp. effectively enhance milk gel texture and reduce syneresis. Notably, EPS from *W. cibaria* SP213 exhibits enhanced interfacial activity under acidic conditions (pH 3), making it particularly suitable for fermented dairy products like yogurt. This EPS contains abundant functional groups (e.g., carboxyl groups), conferring strong hydrophilicity and superior interfacial stabilization [13]. Furthermore, in mixed-culture fermentations, *Weissella* spp. contribute to flavor complexity through heterofermentative metabolism. For instance, Sichuan pickles co-inoculated with *Lactiplantibacillus plantarum* LP067 and *W. cibaria* WC018 showed accelerated acetic and lactic acid production, along with increased volatile flavor compounds (e.g., esters, aldehydes, and ketones) [14]. Due to their prevalence in fermented foods and putative probiotic properties, *Weissella* species are promising starter cultures and potential probiotics. However, their application in yogurt production remains underexplored.

Given the functional potential of *Weissella* spp. in fermented foods—particularly their EPS-producing capacity and flavor-modulating properties—this study investigated the potential of using *W. cibaria* G232 as an adjunct culture to enhance yogurt quality. Building on our laboratory’s prior discovery of its strong EPS production, we systematically evaluated the effects of co-culturing *W. cibaria* G232 with traditional starters (*Lactobacillus delbrueckii* subsp. *bulgaricus* G119 and *Streptococcus thermophilus* Q019) on three critical parameters: physicochemical properties, antioxidant activities, and aromatic profile. To objectively characterize aroma variations, we employed intelligent sensory techniques coupled with gas chromatography-ion mobility spectrometry (GC-IMS). The findings offer novel insights into industrial application of *W. cibaria* G232 in fermented dairy production.

## 2. Materials and Methods

### 2.1. Preparation of Strains

*Weissella cibaria* G232 was isolated from traditional Sichuan fermented pickles. *Lactobacillus delbrueckii* subsp. *bulgaricus* G119 and *Streptococcus thermophilus* Q019 were obtained from commercial yogurt and identified via 16S rRNA sequencing. All strains were cryopreserved at −80 °C in 50% (*v*/*v*) glycerol until use. Prior to experimentation, the cultures were revived by two successive subcultures in de Man, Rogosa, and Sharpe (MRS) broth under aerobic conditions at 30 °C for 24 h.

### 2.2. Yogurt Fermentation

**Figure 1:**
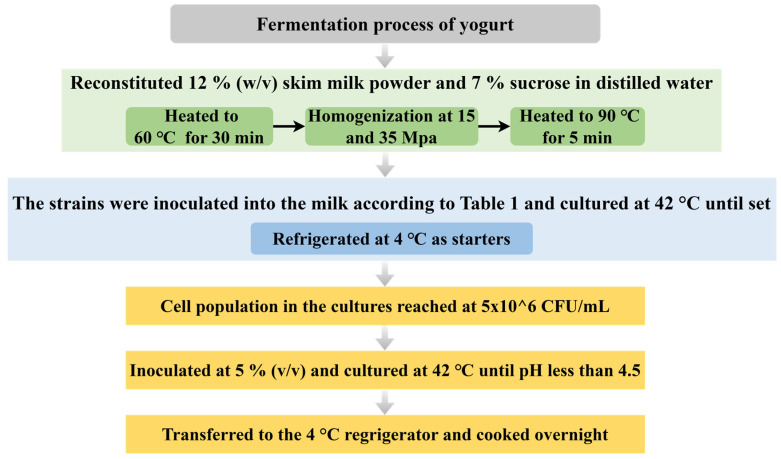
Fermentation process of yogurt.

Initial preliminary experiments confirmed vigorous growth of *W. cibaria* G232, *L. bulgaricus* G119, and *S. thermophilus* Q019 on the MRS medium. Based on these findings, the three strains were inoculated into 10 mL of fresh MRS broth and incubated at 30 °C for 24 h. Cells were harvested through centrifugation (7200× *g*, 30 min, 4 °C, Centrifuge 5920 R, Eppendorf, Hamburg, Germany). Pelleted cells were PBS-washed twice and standardized to 5 × 10^6^ CFU/mL for subsequent yogurt fermentation. Yogurt fermentation was performed according to Figure 1: sterilized milk inoculated with 5% (*v*/*v*) starter culture was incubated at 42 °C. The control group (YC) contained conventional starter cultures (*L. bulgaricus* G119 and *S. thermophilus* Q019, 1:1 ratio). Moreover, yogurt samples were co-inoculated with a traditional starter and a different ratio of *W. cibaria* G232 (3%, Y1, 5%, Y2, and 7%, Y3) while maintaining total inoculum volume at 5% (*v*/*v*), as detailed in Table 1. Fermentation was terminated when the pH decreased below 4.5, after which the coagulated product was immediately refrigerated at 4 °C overnight.

### 2.3. Physicochemical Characteristics of Yogurt

#### 2.3.1. Determination of pH and Titratable Acidity (TA)

The pH of yogurt was measured hourly during the fermentation with a digital pH meter (DGB-402A, Inesa, Shanghai, China). TA of yogurt was determined by titration with sodium hydroxide (NaOH). Specifically, 10 g of yogurt was accurately weighed into tapered bottles and mixed with 20 mL of distilled water. The mixture was titrated with 0.1 mol/L NaOH in the presence of phenolphthalein as an indicator. The TA was calculated using Equation (1).

(1) $$\mathrm{Titratable}\;\mathrm{acidity}=\frac{V\times C\times 100}{m\times 0.1}$$

where C is the concentration of the NaOH standard solution (0.1 mol/L), V is the volume of NaOH (mL), and m is the quantity of the yogurt sample (g).

#### 2.3.2. Determination of Viable Counts

Samples (1 mL) were diluted with 9 mL of normal saline. Serial dilutions were plated onto an MRS agar medium and cultured at 37 °C for 48 h. Colonies were counted on the plate, each containing 30 to 300 colonies.

#### 2.3.3. Determination of Water-Holding Capacity (WHC)

A total of 10 g of yogurt sample was weighed into a 15 mL centrifuge tube and centrifuged at 5000× *g* at 4 °C for 20 min. The supernatant was collected for antioxidant capacity analysis. WHC was calculated following Equation (2).

(2) $$\mathrm{WHC}={\frac{W}{W}}_{0}\times 100$$

where *W* is the weight of precipitation after centrifugation, and *W*_0_ is the weight of the sample.

#### 2.3.4. Determination of Rheological Property

According to the method by Fan et al. [15], the rheological properties of the samples were determined using the HR-1 rheometer (TA-WATERS Ltd., New Castle, DE, USA). The apparent viscosity of the yogurts was measured at 25 °C, with a fixed frequency of 1 HZ and a share rate increasing from 0.1 s^−1^ to 100 s^−1^, followed by a decrease from 100 s^−1^ to 0.1 s^−1^. Modulus (*G*′) and loss modulus (*G*″) were measured at 25 °C under a constant strain fixed at 0.5%; the frequency of scanning ranged from 0.1 to 10 HZ in the shear mode.

#### 2.3.5. Texture Analysis

Hardness, consistency, and cohesiveness of yogurt were measured using a texture analyzer (TA. XT Plus, Stable Micro System, Godalming, Surrey, UK). A cake probe of 35 mm was selected with a test distance of 10 mm and a trigger point of 10.0 g. The pre-test speed was 6 mm/s, and the test speed was 2 mm/s [16]. The yogurt was tested immediately after the removal from the refrigerator, and the temperature in the testing environment was maintained at 25 °C.

### 2.4. Determination of Yogurt’s Antioxidant Activity

A total of 10 g of the yogurt sample was weighed into 15 mL centrifuge tube and centrifuged at 5000× *g*, at 4 °C, for 20 min. The supernatant was mixed with 15 mL of acidified methanol (containing 0.05 mL of hydrochloric acid), thoroughly vortexed, and incubated at −20 °C for 1 h to ensure complete protein precipitation, then centrifuged at 5000× *g* and 4 °C for 10 min.

#### 2.4.1. Determination of ABTS Free Radical Scavenging Ability

The ABTS solution was mixed with 7 mM of 2, 2-azino-bis-(3-ethylbenzo-thiazoline-6-sulfonic acid) diammonium salt and 2.45 mM of potassium persulfate, reacted in darkness overnight. The solution was diluted with 80% ethanol to achieve an absorbance of 0.7 ± 0.02 at 734 nm and stocked until use. Then, 50 μL of the sample was mixed with 1 mL of the diluent ABTS solution and incubated at 25 °C for 30 min in darkness. The absorbance at 734 nm was recorded [17] and the results were calculated following Equation (3).

Scavenging activity (%) = [1 − (A2 − A1)/A0] × 100(3)

where A0 is blank and represents the absorbance of ABTS and ethanol, A1 is control and represents the absorbance of supernatant and ethanol, and A2 represents the absorbance of ABTS and supernatant.

#### 2.4.2. Determination of DPPH Free Radical Scavenging Ability

DPPH (2,2-diphenyl-1-picrylhydrazyl) solution (0.05 mmol/L) was prepared in anhydrous ethanol and stored in light-protected amber vials at 4 °C. For radical scavenging assays, 400 μL test samples were combined with 600 μL DPPH solution (final volume 1 mL) and incubated in darkness at 25 °C for 30 min [17]. Absorbance measurements were conducted at 517 nm (TU-1901 spectrophotometer, Persee General Instrument Co., Ltd., Beijing, China), with anhydrous ethanol serving as the blank control. Radical scavenging activity (%) was calculated according to Equation (4).

Scavenging activity (%) = [1 − (B2 − B1)/B0] × 100(4)

B0 as blank represents the absorbance of DPPH and ethanol, B1 as control represents the absorbance of supernatant and ethanol, and B2 represents the absorbance of DPPH and supernatant.

#### 2.4.3. Ferrous Ion Chelating Ability (FRAP)

Fresh FRAP reagent was mixed with 300 mM acetate buffer, 40 mM 2,4,6-Tripyridyl-s-Triazine (TPTZ) solution, and 20 Mm ferric chloride solution in a volume ratio of 10:1:1. The reaction system consisted of 0.4 mL of the sample and 0.6 mL of the FRAP reagent, incubated in the dark at 37 °C for 10 min. The absorbance of mixture was recorded at 593 nm. FeSO_4_·7H_2_O solutions were used to graph standard curve, and the ferric-reducing ability of the yogurt was expressed as mmol/mL of Fe^2+^ [18].

### 2.5. Determination of Yogurt’s Sensory Properties by Intelligent Technology

#### 2.5.1. Electronic Nose and Tongue Analysis

A commercial electronic nose system (FOX 4000, Alpha MOS, Toulouse, France) equipped with 18 sensor chambers, an injection system, a mass flow controller, and a microcontroller acquisition board, was used to measure the different yogurt samples. The information detected by each sensor is presented in Table 2 [19]. A total of 3 mL of the yogurt sample was put into a 10 mL headspace bottle, then incubated at 40 °C for 5 min and injected into system immediately. The measurement process was maintained for 120 s. Each sample was observed five times. E-tongue System (Astree tongue, Alpha MOS, Toulouse, France) was equipped with 7 different taste sensors, including sourness (AHS), saltiness (CTS), umami (NMS), sweetness (ANS), bitterness (SCS), and two reference electrodes (PKS and CPS). Mixed with 150 mL yogurt sample and 200 mL deionized water, and filtered under normal pressure, the filtrate was collected for electronic tongue analysis [20]. The collection time was set to 120 s, cleaning time to 30 s, and the stirring speed to 60 rpm. Each sample was analyzed five times.

#### 2.5.2. GC-IMS Analysis

The GC-IMS instrument (Flavorspec, G.A.S. Instrument, Munich, Germany) was equipped with an automatic sampling system (CTC Analytics AG, Zwingen, Switzerland) and MXT-WAX capillary column. Accurately weighed 2.0 g sample into a headspace bottle and incubated at 60 °C for 15 min. Then, 500 μL of the headspace sample was injected at 65 °C using a heated syringe, with 99.99% nitrogen as the carrier gas. The injector temperature was maintained at 60 °C, and the GC program flow was set as follows: 2 mL/min for 2 min; 20 mL/min for 8 min; 130 mL/min for 10 min. The headspace samples were eluted and separated at 60 °C [21]. N-ketone (C4–C9) was used as external reference material to calculate the retention index (RI) of volatile compounds. The retention index (RI) and ions’ drift time were compared with the standard in the GC-IMS database for identification.

### 2.6. Statistical Analysis

Data means were calculated from triplicate samples. Significant differences were determined by one-way ANOVA followed by Duncan’s multiple comparison test. Non-parametric correlations between variables were assessed using Spearman’s rank correlation analysis, with correlation coefficients and *p*-values calculated via IBM SPSS Statistics 27.0 (SPSS Inc., Chicago, IL, USA). PCA and clustering heatmap visualizations were generated using Origin 2022 (OriginLab, Northampton, MA, USA). All statistical analyses, including ANOVA, post hoc tests, and Spearman correlation, were conducted using SPSS.

## 3. Results

### 3.1. Changes in Acidity of Yogurt During Fermentation

**Figure 2:**
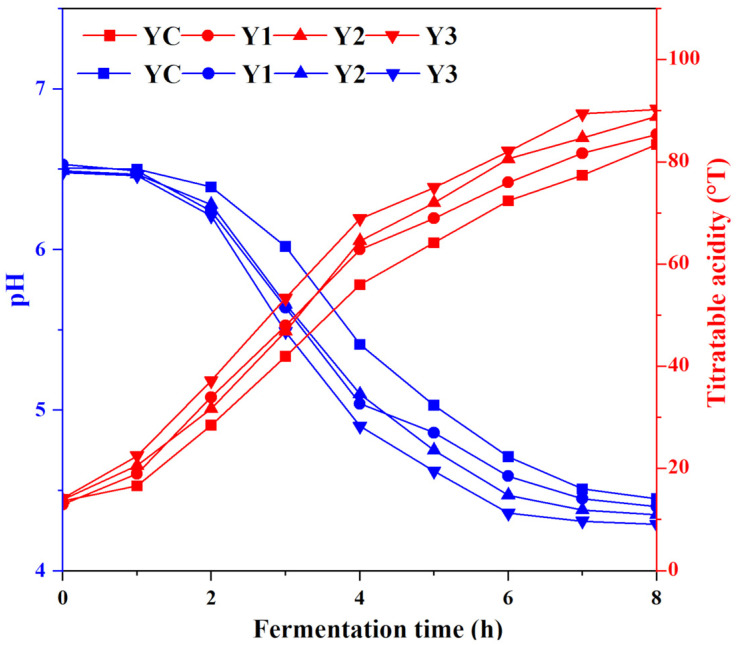
Changes in the pH and titratable acidity during fermentation. (Note: YC: *L. bulgaricus* G119: *S. thermophilus* Q019 (1:1); Y1: YC + 3% *W. cibaria* G232; Y2: YC + 5% *W. cibaria* G232; Y3: YC + 7% *W. cibaria* G232.).

Figure 2 exhibited the changes in pH and TA values of different groups. Before fermentation, the pH of milk was 6.5. In the first 4 h during fermentation, the acidity of yogurt drops dramatically, which corresponds to a significant increase in TA values. After that, the acidity levels among the four groups showed little variation. At 6 h, both Y2 and Y3 groups reached the end point, and their pH values were 4.47 and 4.36, respectively—lower than those of the control groups (4.59). Compared to the control group, 5% and 7% *W. cibaria* G232 addition reduced the fermentation time from 7 h to 6 h.

### 3.2. Changes in Viable Counts and WHC of Yogurt During Fermentation

**Figure 3:**
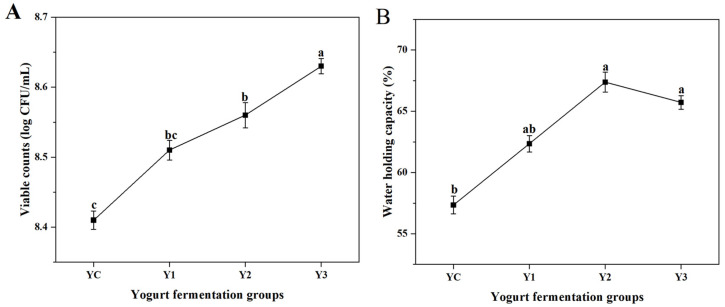
Changes in the viable counts (**A**) and water holding capability (**B**) in different yogurt groups. (Note: YC: *L. bulgaricus* G119: *S. thermophilus* Q019 (1:1); Y1: YC + 3% *W. cibaria* G232; Y2: YC + 5% *W. cibaria* G232; Y3: YC + 7% *W. cibaria* G232. Averages labeled with different lowercase letters indicate a significant difference (*p* < 0.05).).

The changes in viable counts and water holding capacity of the fermented milk are shown in Figure 3A and Figure 3B, respectively. With the incremental elevation in the proportion of *W. cibaria* G232, the viable counts in the yogurt increased significantly (*p* < 0.05), demonstrating a dose-dependent relationship with the proportion of *W. cibaria* G232. The experimental group Y3 demonstrated a marked increase from 8.41 log CFU/mL in the control group (YC) to 8.63 log CFU/mL (*p* < 0.05). Concurrently, water holding capacity (WHC) exhibited a concentration-dependent enhancement pattern, rising from 57.35% in YC to 67.38% in Y2 (*p* < 0.05). Although WHC experienced a marginal reduction to 65.72% at the maximal supplementation level (7% G232), this value remained significantly elevated compared to the control (*p* < 0.05). The enhanced WHC values indicated a compact tissue state and improved the retention of aromas. These findings collectively demonstrate that *W. cibaria* G232 functions as an effective co-starter culture, simultaneously enhancing both microbial viability and water holding capability in yogurt fermentation systems.

### 3.3. Rheological Properties of Yogurt

**Figure 4:**
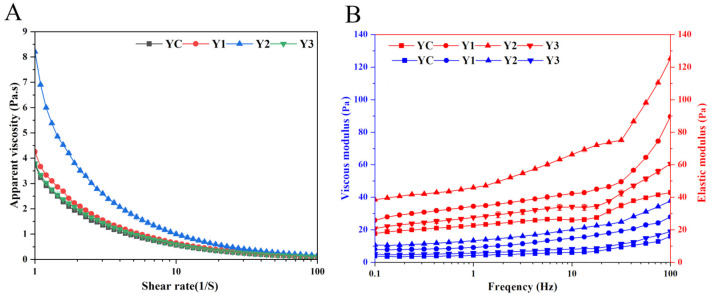
Changes in apparent viscosity (**A**), the viscous modulus *G*″ and elastic modulus *G*′ (**B**) of yogurts supplemented with different proportions of *W. cibaria* G232. (Note: YC: *L. bulgaricus* G119: *S. thermophilus* Q019 (1:1); Y1: YC + 3% *W. cibaria* G232; Y2: YC + 5% *W. cibaria* G232; Y3: YC + 7% *W. cibaria* G232).

The shear stability of yogurt was assessed in Figure 4A,B. The apparent viscosity of all yogurt showed the same change trends and decreased with the increasing of shear rate during the scan, until it finally stabilized. The apparent viscosity of yogurt co-fermented with *W. cibaria* G232 and traditional starter was higher than control group YC; the highest apparent viscosity was observed in Y2. However, when the addition of *W. cibaria* G232 reached 7% (Y3), the effect on the viscosity of yogurt is not obvious.

*G*′ (elastic module) and *G*″ (viscous module) of the four groups of yogurts showed the same variation trends during test processing. *G*′ > *G*″ on all frequency indicated that yogurt has solid-like characteristics; both indicators increased gradually when frequency increased from 0.1 to 100 Hz. On the lower frequency (0.1–10 Hz), *G*′ and *G*″ increased slowly; however, the higher frequency (>10 Hz) also increased the rise rate of *G*′ and *G*″. Furthermore, the value of *G*′ and *G*″ of yogurt with *W. cibaria* G232 (Y1, Y2 and Y3) was higher than control group YC. When the addition of *W. cibaria* G232 was 5%, the highest values of *G*′ and *G*″ were observed in the Y2 group.

### 3.4. Texture Change in Yogurt

Texture properties of yogurt are shown in Table 3. Firmness serves as an indicator of coagulation quality and resistance to deformation, and cohesiveness serves as an index to characterize the structural integrity and smoothness of yogurt. The most favorable textural properties were observed at a 5% addition of *W. cibaria* G232 (Y2), with the yogurt exhibiting the highest levels of firmness, consistency and cohesiveness. When the addition of G232 reached 7%, the three indicators of texture showed varying degrees of decline; however, the three groups Y1, Y2, and Y3 still showed improvement compared to the control group YC. Table 3 showed that the yogurt co-fermented with *W. cibaria* G232 had comprehensive advantages in firmness, consistency, and cohesiveness compared to the control group, indicating that *W. cibaria* G232 could greatly improve the texture and quality of the yogurt.

### 3.5. Antioxidant Activity Change in Yogurt

**Figure 5:**
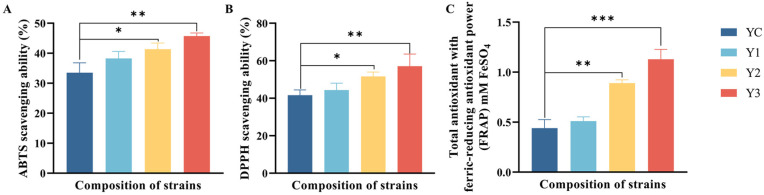
Antioxidant activity of yogurt with different proportions of *W. cibaria* G232. (Note: (**A**) is the ABTS scavenging ability; (**B**) is the DPPH scavenging ability; (**C**) is the total antioxidant with ferric-reducing antioxidant power; where * stands for *p* < 0.05, ** stands for *p* < 0.01, *** stands for *p* < 0.001. YC: *L. bulgaricus* G119: *S. thermophilus* Q019 (1:1); Y1: YC + 3% *W. cibaria* G232; Y2: YC + 5% *W. cibaria* G232; Y3: YC + 7% *W. cibaria* G232.).

The addition of *W. cibaria* G232 significantly enhanced the antioxidant activity of yogurt, which also demonstrated a dose-dependent relationship with *W. cibaria* G232 in Figure 5. When the addition of *W. cibaria* G232 was at 7%, the highest antioxidant activity of yogurt was observed. Compared to the control group, the ABTS radical scavenging ability significantly increased from 33.54 ± 3.26% to 45.74 ± 1.03% (*p* < 0.01), and the value of DPPH radical scavenging ability increased from 44.37 ± 3.69% to 57.08 ± 6.48% (*p* < 0.01). Additionally, the FRAP value of yogurt significantly increased from 0.44 ± 0.09 to 1.13 ± 0.10 mM at the 7% addition of G232 (*p* < 0.001), showing a remarkable improvement in antioxidant activity compared to control group YC (*p* < 0.001).

### 3.6. E-Tongue and E-Nose Analysis of Yogurt

**Figure 6:**
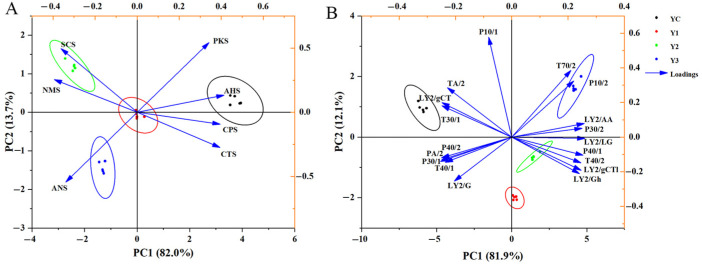
PCA model and loading plot based on the response data of E-tongue (**A**) and E-nose (**B**) of different yogurt groups. (Note: YC: *L. bulgaricus* G119: *S. thermophilus* Q019 (1:1); Y1: YC + 3% *W. cibaria* G232; Y2: YC + 5% *W. cibaria* G232; Y3: YC + 7% *W. cibaria* G232.).

The electronic tongue analyzer was equipped with seven sensors. Sensors of PKS, ANS, and SCS showed significant responses when analyzing the four different groups of yogurts. Figure 6A presents the results of PCA, which was based on the response values of different sensors. The horizontal axis represents the contribution rate of PC1 (82.0%), meanwhile the vertical axis represents the contribution rate of PC2 (13.7%). The cumulative contribution rate of the two principal components reached 95.7%, indicating an effective dimensionality reduction while preserving essential sample characteristics. As shown in the graphic, the two principal components retained the main characteristics of the samples, with the horizontal axis (PC1) contributing more significantly than the vertical axis (PC2). The position of the YC sample was further along the PC1 axis compared to the other groups, suggesting statistically significant organoleptic differentiation in taste profile parameters.

The electronic nose analyzer was equipped with 18 different odor sensors to identify the flavor profile of fermented milks. Figure 6B shows that the results of PCA, YC, Y1, Y2, and Y3 were distinguished well by the E-nose, indicating that fermentation generated the distinct, characteristic, gas flavor profile. The contribution rate of horizontal axis (PC1) interpreted 81.9% of the data information, while the vertical axis (PC2) accounted for 12.1%. From a horizontal coordinate perspective, YC was more distant from the other samples, which indicates that YC differs greatly from the other samples in terms of PC1. In addition, Y1 and Y2 were closer together, indicating similar odor compositions. According to the loading diagram, the sensors of P10/1 and T70/2 contributed more significant values to the PCA. These results demonstrate that the sensory characteristics of the yogurt can be effectively differentiated using the E-nose and E-tongue. In the E-nose sensor response profiles, Sensors LY2/LG and P30/2 exhibited the highest loadings on PC1, suggesting that the volatile organic compounds detected by these sensors are strongly associated with the floral and delicate aromatic profile of fermented yogurt.

### 3.7. Volatile Aroma Compound Analysis of Yogurt (GC-IMS)

**Figure 7:**
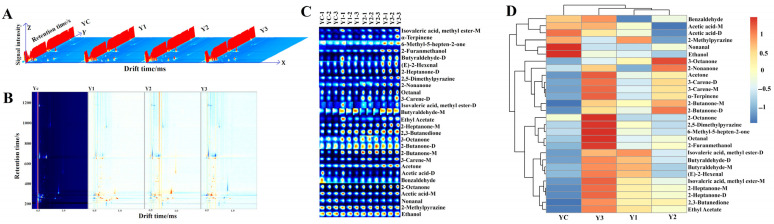
Observation results of GC-IMS on fermented yogurts with different proportions of *W. cibaria* G232. (Note: (**A**) is the three-dimensional representation; (**B**) is the bird’s-eye view representation, with spectra from the control group employed as a reference and the corresponding spectra from yogurt fermented with *W. cibaria* G232 represented as differences from the control group YC; (**C**) is the representation of ion migration spectra, where the ions are numbered and then listed as gallery plots, in which the color was brighter, the content was higher; (**D**) is the clustering map of yogurts with different proportions of *W. cibaria* G232 metabolites. YC: *L. bulgaricus* G119: *S. thermophilus* Q019 (1:1); Y1: YC + 3% *W. cibaria* G232; Y2: YC + 5% *W. cibaria* G232; Y3: YC + 7% *W. cibaria* G232.).

The volatile metabolic profiles of fermented yogurts with different concentration of *W. cibaria* G232 were analyzed using GC-IMS. Figure 7A shows the 3D visualization plot where the transverse axis represents the ion migration time, the longitudinal axis represents the gas chromatography retention time, and the vertical axis refers to the intensity of the signal peak.

In the 3D topographic map, the visualization patterns of different samples were similar, but the peak heights of different compounds varied slightly. When yogurt was fermented without the *W. cibaria* G232 used as the spectral background, the other samples exhibited distinct red and blue spots in the spectrogram (Figure 7B). The red spots represented higher concentration of volatile aroma compounds compared to the control group YC, while the blue spots represented lower concentrations. Compared to the control group YC, the yogurt supplemented with *W. cibaria* G232 showed increased diversity and abundance of volatile aroma substances, indicating that *W. cibaria* G232 facilitated the formation and accumulation of volatile aroma compounds.

Figure 7C showed the fingerprint of the volatile aroma substances in fermented yogurt, which exhibited the influence of the different *W. cibaria* G232 concentrations on these compounds. A total of twenty-two aroma compounds were detected in the four different yogurt groups, including six aldehydes, seven ketones, two alcohols, one acid, two esters, and four other aroma substances. As shown in Table 4, compared to control group YC, the content of acetone, 2-heptanone, 2-butanone, 2,3-butanedione, ethyl acetate, butyraldehyde, and other substance increased significantly in yogurt co-fermented with *W. cibaria* G232 and traditional starter cultures (*p* < 0.05). On the contrary, the content of nonanal and ethanol, which were the main aroma substances in the control group YC, significantly decreased (*p* < 0.05). The results showed that with the addition of different concentrations of *W. cibaria* G232, the content of the volatile aroma gradually increased, and when the concentration of *W. cibaria* G232 reached 7%, the content of the aroma substances reached maximum.

The volatile aroma substances in yogurt are primarily produced through the degradation of milk fat and bioconversion by lactic acid bacteria. The heat map was generated based on the relative contents of volatile aroma substances in the four different yogurt groups, and cluster analysis was also performed in Figure 7D. The yogurt samples were divided into two clusters based on their volatile aroma profiles. YC formed one cluster and Y1, Y2, and Y3 were gathered in other clusters. The heatmap visually represents the differences in volatile aroma substance profiles between YC and the other groups. Variations in the contents of different aroma substances contributed to the distinct aroma differences in YC and Y3, which aligns with the results of the PCA obtained from the E-nose analysis.

### 3.8. Correlation Between E-Nose and GC-IMS

**Figure 8:**
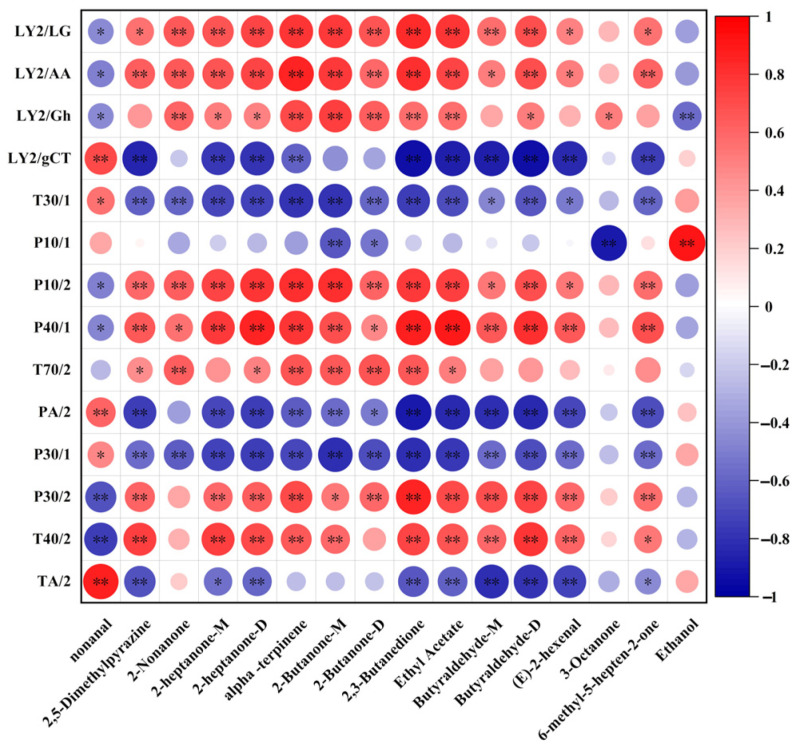
Spearman’s correlation heatmap of the significantly altered volatile compound levels and the E-nose sensor responses. (Colors represent the correlation coefficients, with red and blue indicating positive and negative correlation, respectively. Asterisks * and ** stand for significance at *p* < 0.05 and *p* < 0.01, respectively).

As illustrated in Figure 8, the sensors LY2/LG, P10/2, and LY2/AA, associated with coffee, fruity, and special aromas, exhibited a significant positive correlation with volatile compounds of 2-heptanone, α-terpinene, 2-butanone, 2,3-butanedione, and ethyl acetate (*p* < 0.01). These compounds were present in higher concentration in yogurts co-fermented with *W. cibaria* G232. On the contrary, sensors LY2/gCT, PA/2 showed a significant positive correlation with nonanal, while sensors p40/1 was correlated with ethanol. Nonanal and ethanol were found in greater quantities in control group fermented without *W. cibaria* G232 (YC). This finding indicates that both E-nose and GC-IMS are effective in distinguishing aroma compounds in fermented milk at different levels. Moreover, the yogurt co-fermented with *W. cibaria* G232 exhibited a greater richness in aroma substances, highlighting its dominance in aroma profile development.

## 4. Discussion

Fermented dairy products are widely consumed around the world, with LAB playing a crucial role in the fermentation of milk. Previous studies have highlighted several advantages of *Weissella* as a fermenting agent [22], such as reducing fermentation time and inhibiting the growth of pathogenic microorganisms through acid production [23]. Additionally, *Weissella* has been shown to improve the quality and flavor of fermented products by breaking down proteins, lactose, and fats [24]. Although *Weissella* spp. are recognized as excellent probiotics with significant potential in food fermentation, their application in yogurt production remains underexplored, and previous studies mainly focused on their effects on yogurt texture, rheological properties, and bacterial viability. In this study, gas chromatography-ion mobility spectrometry (GC-IMS) and intelligent sensory technologies (E-nose and E-tongue) were employed to systematically investigate the dynamic changes in volatile aroma compounds during yogurt fermentation with *W. cibaria* G232. Furthermore, the impact of co-fermentation with *W. cibaria* G232 on the antioxidant properties of yogurt was comprehensively evaluated. This research emphasizes three critical aspects (physicochemical properties, antioxidant activity, and aromatic profiles) of yogurt quality enhancement, providing valuable theoretical insights and experimental data to support the application of *W. cibaria* G232 as a co-starter in yogurt production.

Coagulation time and acidification rate of yogurt is significantly influenced by pH and titratable acidity (TA) values. In this study, the addition of 5%–7% *W. cibaria* G232 (Y2 and Y3) shortened the fermentation time from 7 h to 6 h compared to the control group YC. The reduction was attributed to the accelerated acidification process, driven by the synergistic interaction between *W. cibaria* G232 and the traditional starters (*L. bulgaricus* G119 and *S. thermophilus* Q019). *Weissella cibaria*, as a typical heterofermentative bacterium, produces lactic acid, CO_2_, and acetate via the hexose monophosphate and phosphoketolase pathways [25]. *L. bulgaricus* and *S. thermophilus* are homofermentative lactic acid bacteria; however, the available literature indicates that the coexistence of homo- and hetero-lactic bacteria can accelerate the fermentation process [13]. Notably, the pH decline was most pronounced during the first four hours of fermentation; after that, the acidity levels among the four groups showed little variation. This phenomenon may be due to the promoting effect of *W. cibaria* G232 on the growth environment of *Streptococcus thermophilus* Q019. During the initial stage of fermentation, the rapid growth and acid production by *S. thermophilus* significantly shortened the fermentation time. These results align with the findings of Zou et al. [24], who observed that co-fermentation with *W. confusa* SW1 reduced the curd time of yogurt and promoted the growth of *S. thermophilus* ST51. Moreover, synergistic effects on glycolysis and lactate production may further enhance the fermentation process.

Water-holding capacity and rheological and texture properties are determined by the network arrangement structure of yogurt and influenced by the manufacturing process [26]. In this study, co-fermentation of traditional starters with *W. cibaria* G232 promoted protein aggregation in milk, resulting in higher WHC and apparent viscosity in the yogurt. At a 5% addition level of *W. cibaria* G232, both the elastic modulus (*G*′) and viscous modulus (*G*″) reached their maximum, indicating optimal stability and smoothness of the yogurt. In addition, the co-fermentation of traditional starters with *W. cibaria* G232 improved the hardness, consistency, and cohesiveness of the yogurt to varying degrees. These quality enhancements may be attributed to the exopolysaccharides (EPS) produced by *W. cibaria* G232 during fermentation. Our previous studies have shown that *W. cibaria* G232 has a strong ability to produce extracellular polysaccharides (EPS). Also, the literature showed that the *Weissella* species, including *W. cibaria*, *W. confusa*, and *W. hellenica*, are well-known EPS producers [27]. The hydroxyl and negatively charged groups in the side chains of EPS can bind water molecules, thereby reducing whey separation and enhancing the stability of yogurt [28]. Moreover, the interaction between EPS and casein (CAS) via intermolecular hydrogen bonding and electrostatic and hydrophobic contacts enhances the charge density and electrostatic potential of molecules. This improves the viscidity and elasticity of the fermented system [29], which is consistent with the findings of our study.

Even though the positive effects of EPS on fermented products are well-documented, excessive EPS in a yogurt fermentation system can strengthen the steric barrier [30], reducing electrostatic interactions between particles and potentially weakening the stability of the fermented system. The finding is also consistent with our results. The addition of *W. cibaria* G232 at a concentration of 5% resulted in optimal fermentation outcomes, including improved texture and rheological properties, whereas a concentration of 7% was less effective, likely due to the steric hindrance caused by excessive EPS production. Antioxidant potential (AP) is one of the most important nutritional properties of yogurt, as it not only extends the shelf life but also helps to prevent oxidative damage. ABTS and DPPH are widely used to evaluate free radical scavenging capacity due to their rapid, simple, and cost-effective feature. FRAP assay measures the reducing power of antioxidants by quantifying the reduction in the Fe^3+^-TPTZ complex to Fe^2+^-TPTZ, resulting in strong absorption at specific wavelengths [31]. Our results reveal that mixed fermentation significantly enhanced the antioxidant activity of yogurt (*p* < 0.05) in a dose-dependent manner, with respect to the concentration of *W. cibaria* G232. *Weissella* spp. are well known for their antioxidant properties, particularly from the exopolysaccharides (EPS) they produce. These EPS hydrolyze in acidic environments, exposing active hemiacetal hydroxyl groups that transfer electrons to free radicals, facilitating the conversion of free radicals into stable compounds and reducing oxidative damage [32]. Moreover, the antioxidant capacity of fermented products also closely relates to the viable cell counts. Simionescu and Petrovici [33] demonstrated that the antioxidant activity in fermented media increased with the number of CFU/mL of *W. confusa* PP29, which is consistent with our results. The addition of *W. cibaria* G232 at 7% resulted in the highest viable cell count (8.63 log CFU/mL) among the four groups, accompanied by the highest ABTS and DPPH radical scavenging activities and ferric-reducing antioxidant power, which can be attributed to the synergistic effects of various antioxidant components, including casein, peptides, antioxidant enzymes [34].

E-nose and GC-IMS were employed to distinguish and analyze the aromatic profiles of fermented yogurt from two different dimensions. Previous studies have shown that the E-nose, equipped with a variety of odor sensors, enables it to effectively extract and reflect the overall information of flavor compounds [35]. In comparison, GC-IMS allows for both qualitative and quantitative analysis of volatile flavor compounds, providing more precise and detailed results. To obtain comprehensive flavor information, we combined both techniques in this study. The generation of volatile compounds in yogurt is highly dependent on the strains used during fermentation. According to previous studies, mixed fermentation tends to be more conducive to the production and accumulation of aroma substances due to the synergistic interactions between different microbial species [8,36]. The aroma of fermented dairy products is mainly derived from microbial transformation and chemical reactions, including glycolysis, proteolysis, and lipolysis [37]. The aromatic profile of yogurt is the result of the combined effects of various aroma compounds, including alcohols, acids, aldehydes, ketones, and esters. However, not all the aroma compounds in yogurt play a decisive role. In our study, the content of ethanol and nonanal in yogurt co-fermented with *W. cibaria* G232 significantly decreased compared to the control group (*p* < 0.05). Conversely, the content of ketones, including acetone, 2-heptanone, 2-butanone, and 2- and 3-butanedione was significantly elevated (*p* < 0.05). Ethanol probably provides a complementary flavor to yogurt accrued through decomposition of glucose and amino acids [38]. Nonanal is characterized by a sweet, floral, citrus, and fatty aroma [39]. Both compounds were more abundant in the control group and served as primary aroma compounds in yogurt. In this study, the concentrations of six ketone compounds in yogurt increased variably after co-fermentation. Ketones, primarily formed through the oxidation of free fatty acids and the degradation of amino acids, can directly influence the aroma of fermented dairy products [40]. 2,3-butanedione, an important aroma compound, is produced during the fermentation of citrate in milk. The increase in 2,3-butanedione enhanced the rate of citrate metabolism and contributed to a buttery flavor [41]. Additionally, 2-heptanone can help mitigate the pungent flavor in yogurt [42]. Other compounds such as acetone and butanedione considerably provide a pleasant floral, fruity, and creamy flavor [43]. The incorporation of *W. cibaria* G232 potentially augmented the metabolic pathways associated with fatty acids and amino acids, culminating in the biosynthesis of a substantial quantity of ketones, which contributed notably to positive aromatic profiles such as fruity, sweet, milky, and creamy-cheesy aromas, thereby imparting the complexity and balance of the flavor profile. Moreover, six different aldehyde aroma compounds were also detected. Butyraldehyde, which offers a cocoa-like flavor, showed significantly increased accumulation in the co-fermentation yogurt compared to the control group (*p* < 0.05). Hexanal and heptanal, which provide grassy aromas [44], were present in low concentrations across the fermentation groups without significant differences (*p* > 0.05). Although each flavor compound constitutes a recognizable aroma, the flavor of yogurt is determined by the balanced mixture of key volatile compounds. The synergistic effects of multiple volatile flavor compounds contribute to the production of superior yogurt products. Yogurt fermented with traditional strains contained higher levels of nonanal and ethanol, contributing to a rich and balanced oily flavor. In contrast, the co-fermented yogurt with *W. cibaria* G232 increased the concentration of 2,3-butanedione, enhancing the buttery flavor. Additionally, the accumulation of acetone, butanone, and ethyl acetate enhanced the complexity of the aroma profile, with distinct layers of fruity, creamy, and cocoa notes. The differences in the concentrations of typical volatile compounds were consistent with the results obtained from the E-nose analysis. There was a significant improvement in the aroma of yogurt co-fermented with *W. cibaria* G232, as evidenced by both intelligent sensory techniques and GC-IMS. These findings highlight the potential of mixed fermentation to enhance the aroma profile of yogurt through the synergistic effects of microbial metabolism.

## 5. Conclusions

In conclusion, this study investigated the effects of co-fermentation using *W. cibaria* G232 and traditional starter. The results demonstrate that *W. cibaria* G232 exhibits a positive effect on the physicochemical properties, antioxidant capacities, and volatile profiles of fermented milk. Co-fermentation with *W. cibaria* G232 resulted in a shorter fermentation duration, which was attributed to the synergistic acidification by *Weissella* spp. Furthermore, *W. cibaria* G232 and traditional starter enhanced the vitality of the strains through species interactions. The metabolites produced by *W. cibaria* G232 enhanced stability of yogurt, contributing to improvements in water-holding capacity, rheological properties, and texture. Antioxidant potential of yogurt was significantly enhanced by co-fermentation, showing a dose-dependent relationship with the concentration of *W. cibaria* G232. Intelligent sensory technology combined with GC-IMS revealed that compared to the control group, co-fermentation with *W. cibaria* G232 notably altered the accumulation of crucial aroma compounds, including aldehydes, ketones, and alcohols in the fermented milk. Optimized at 5% *W. cibaria* G232 supplementation, the yogurt exhibited superior quality through balanced physicochemical properties, enhanced antioxidant capacity, and refined aromatic profiles. Excessive supplementation (7%) triggered EPS overproduction, destabilizing casein gels and compromising structural integrity. Overall, the results support the quality improvement of yogurt with the addition of *W. cibaria* G232. However, the specific mechanism underlying the molecular mechanisms of interspecies interactions between *W. cibaria* G232 and traditional starters—particularly through multi-omic approaches (e.g., metabolomics and proteomics) to map cross-species metabolic networks associated with this enhancement—remain to be further explored.

## Figures and Tables

**Table 1:** Combinations of strains.

Group	Different Proportional Combinations
YC	*L. bulgaricus* G119: *S. thermophilus* Q019 is 1:1
Y1	YC + *W. cibaria* G232 (3%)
Y2	YC + *W. cibaria* G232 (5%)
Y3	YC + *W. cibaria* G232 (7%)

**Table 2:** Performance description of the E-nose sensors.

Sensors	Performance Description
LY2/LG	Sensitive to oxidizing gas
LY2/G	Sensitive to ammonia, carbon monoxide
LY2/AA	Sensitive to ethanol
LY2/Gh	Sensitive to ammonia/organic amines
LY2/gCT1	Sensitive to hydrogen sulfide
LY2/gCT	Sensitive to propane/butane
T30/1	Sensitive to organic solvents
P10/1	Sensitive to hydrocarbons
P10/2	Sensitive to methane
P40/1	Sensitive to fluorine
T70/2	Sensitive to aromatic compounds
PA/2	Sensitive to ethanol, ammonia/organic amines
P30/1	Sensitive to polar compounds (ethanol)
P40/2	Sensitive to heteroatom/chloride/aldehydes
P30/2	Sensitive to alcohol
T40/2	Sensitive to aldehydes
T40/1	Sensitive to chlorinated compounds
TA/2	Sensitive to air quality

**Table 3:** Texture changes in yogurt supplemented with different proportions of G232.

Parameters	YC	Y1	Y2	Y3
Firmness (g)	212.85 ± 4.87 ^c^	236.16 ± 7.31 ^b^	259.12 ± 4.44 ^a^	245.91 ± 3.82 ^b^
Consistency (g·s)	936.02 ± 15.26 ^d^	1778.28 ± 13.84 ^b^	1986.75 ± 18.13 ^a^	1324.94 ± 8.16 ^c^
Cohesiveness (g)	−56.06 ± 3.94 ^b^	−46.95 ± 4.73 ^ab^	−40.41 ± 4.82 ^a^	−50.18 ± 7.22 ^ab^

Note: YC: *L. bulgaricus* G119: *S. thermophilus* Q019 (1:1); Y1: YC + 3% *W. cibaria* G232; Y2: YC + 5% *W. cibaria* G232; Y3: YC + 7% *W. cibaria* G232. The data are presented as average values ± SD (n = 3). Averages featuring varying lowercase letters within the same row show a notable difference (*p* < 0.05).

**Table 4:** Molecules’ peak areas (mean ± sd) characterized by GC–IMS in fermented yogurt.

Volatile Flavor Compounds	CAS Registry Number	Molecule Formula	YC	Y1	Y2	Y3
Acetic acid-M	C64197	C_2_H_4_O_2_	4.25 × 10^3^ ± 4.79 × 10^2 a^	3.96 × 10^3^ ± 6.94 × 10^2 a^	3.50 × 10^3^ ± 4.40 × 10^2 a^	4.27 × 10^3^ ± 2.43 × 10^2 a^
Acetic acid-D	C64197	C_2_H_4_O_2_	3.85 × 10^2^ ± 1.04 × 10^2 a^	3.12 × 10^2^ ± 1.06 × 10^2 a^	2.50 × 10^2^ ± 43.02 ^a^	3.44 × 10^2^ ± 40.66 ^a^
Nonanal	C124196	C_9_H_18_O	3.81 × 10^3^ ± 2.34 × 10^2 a^	2.54 × 10^3^ ± 1.81 × 10^2 b^	2.84 × 10^3^ ± 3.20 × 10^2 b^	2.58 × 10^3^ ± 49.62 ^b^
2,5-Dimethylpyrazine	C123320	C_6_H_8_N_2_	9.17 × 10^2^ ± 51.61 ^b^	1.03 × 10^3^ ± 1.32 × 10^2 b^	9.24 × 10^2^ ± 66.22 ^b^	1.29 × 10^3^ ± 58.32 ^a^
2-Octanone	C111137	C_8_H_16_O	9.25 × 10^3^ ± 9.17 × 10^2 a^	8.89 × 10^3^ ± 1.56 × 10^2 a^	8.92 × 10^3^ ± 7.39 × 10^2 a^	1.04 × 10^4^ ± 3.02 × 10^2 a^
2-Methylpyrazine	C109080	C_5_H_6_N_2_	8.74 × 10^3^ ± 3.31 × 10^2 a^	8.78 × 10^3^ ± 3.46 × 10^2 a^	8.12 × 10^3^ ± 2.91 × 10^2 a^	8.31 × 10^3^ ± 47.33 ^a^
Octanal	C124130	C_8_H_16_O	8.51 × 10^2^ ± 1.33 × 10^2 a^	8.29 × 10^2^ ± 1.37 × 10^2 a^	8.04 × 10^2^ ± 95.03 ^a^	8.63 × 10^2^ ± 27.26 ^a^
2-Nonanone	C821556	C_9_H_18_O	1.11 × 10^3^ ± 36.43 ^ab^	1.05 × 10^3^ ± 98.29 ^b^	1.33 × 10^3^ ± 1.26 × 10^2 a^	1.27 × 10^3^ ± 20.43 ^ab^
Acetone	C67641	C_3_H_6_O	4.01 × 10^2^ ± 20.15 ^c^	4.54 × 10^2^ ± 37.53 ^bc^	4.90 × 10^2^ ± 22.22 ^b^	5.48 × 10^2^ ± 15.08 ^a^
2-Heptanone-M	C110430	C_7_H_14_O	1.16 × 10^3^ ± 98.33 ^b^	1.38 × 10^3^ ± × 1.41 × 10^2 ab^	1.33 × 10^3^ ± 1.22 × 10^2 ab^	1.51 × 10^3^ ± 20.52 ^a^
2-Heptanone-D	C110430	C_7_H_14_O	2.75 × 10^2^ ± 34.11 ^b^	3.63 × 10^2^ ± 59.44 ^ab^	3.34 × 10^2^ ± 38.18 ^ab^	4.21 × 10^2^ ± 9.48 ^a^
3-Carene-M	C13466789	C_10_H_16_	5.09 × 10^2^ ± 1.06 × 10^2 a^	5.94 × 10^2^ ± 1.74 × 10^2 a^	7.13 × 10^2^ ± 1.64 × 10^2 a^	8.08 × 10^2^ ± 1.44 × 10^2 a^
3-Carene-D	C13466789	C_10_H_16_	1.81 × 10^2^ ± 43.73 ^a^	2.18 × 10^2^ ± 68.89 ^a^	2.88 × 10^2^ ± 90.04 ^a^	3.24 × 10^2^ ± 82.34 ^a^
α-Terpinene	C99865	C_10_H_16_	1.40 × 10^2^ ± 19.07 ^b^	1.67 × 10^2^ ± 46.86 ^ab^	2.16 × 10^3^ ± 50.74 ^ab^	2.56 × 10^2^ ± 53.92 ^a^
2-Butanone-M	C78933	C_4_H_8_O	9.56 × 10^2^ ± 91.59 ^c^	1.79 × 10^3^ ± 1.76 × 10^2 b^	2.21 × 10^3^ ± 1.45 × 10^2 a^	2.09 × 10^3^ ± 24.04 ^a^
2-Butanone-D	C78933	C_4_H_8_O	5.21 × 10^2^ ± 21.61 ^c^	5.52 × 10^2^ ± 51.26 ^b^	5.97 × 10^2^ ± 26.89 ^a^	5.78 × 10^2^ ± 11.93 ^a^
Ethanol	C64175	C_2_H_6_O	2.79 × 10^3^ ± 13.18 ^a^	2.47 × 10^3^ ± 7.23 ^b^	2.41 × 10^3^ ± 28.16 ^c^	2.51 × 10^3^ ± 14.21 ^b^
2,3-Butanedione	C431038	C_4_H_6_O_2_	1.98 × 10^3^ ± 80.30 ^c^	3.50 × 10^3^ ± 1.18 × 10^2 b^	3.50 × 10^3^ ± 3.04 × 10^2 b^	4.20 × 10^3^ ± 98.20 ^a^
Ethyl Acetate	C141786	C_4_H_8_O_2_	1.00 × 10^2^ ± 6.51 ^b^	2.50 × 10^2^ ± 85.55 ^a^	2.10 × 10^2^ ± 36.09 ^a^	3.02 × 10^2^ ± 7.57 ^a^
Butyraldehyde-M	C123728	C_4_H_8_O	2.10 × 10^2^ ± 11.29 ^b^	3.60 × 10^2^ ± 39.97 ^a^	2.24 × 10^2^ ± 16.91 ^b^	3.78 × 10^2^ ± 7.81 ^a^
Butyraldehyde-D	C123728	C_4_H_8_O	81.01 ± 7.85 ^b^	2.30 × 10^2^ ± 47.81 ^a^	1.31 × 10^2^ ± 5.61 ^b^	2.57 × 10^2^ ± 5.51 ^a^
2-Furanmethanol	C98000	C_5_H_6_O_2_	1.53 × 10^3^ ± 1.09 × 10^2 c^	1.74 × 10^3^ ± 2.23 × 10^2 bc^	1.86 × 10^3^ ± 91.15 ^b^	2.38 × 10^3^ ± 6.59 ^a^
Benzaldehyde	C100527	C_7_H_6_O	2.21 × 10^2^ ± 58.58 ^a^	1.94 × 10^2^ ± 16.22 ^a^	2.09 × 10^2^ ± 10.15 ^a^	2.26 × 10^2^ ± 8.72 ^a^
(E)-2-Hexenal	C6728263	C_6_H_10_O	2.04 × 10^2^ ± 11.26 ^b^	2.38 × 10^2^ ± 17.68 ^a^	2.08 × 10^3^ ± 10.04 ^b^	2.45 × 10^2^ ± 5.51 ^a^
Isovaleric acid, methyl ester-M	C556241	C_6_H_12_O_2_	1.80 × 10^2^ ± 23.57 ^a^	2.68 × 10^2^ ± 97.63 ^a^	2.31 × 10^2^ ± 48.19 ^a^	3.21 × 10^2^ ± 61.19 ^a^
Isovaleric acid, methyl ester-D	C556241	C_6_H_12_O_2_	70.98 ± 3.09 ^a^	90.02 ± 24.14 ^a^	76.94 × 10^3^ ± 7.79 ^a^	89.10 ± 12.06 ^a^
3-Octanone	C106683	C_8_H_16_O	1.36 × 10^2^ ± 11.26 ^ab^	1.51 × 10^2^ ± 3.68 ^ab^	1.67 × 10^2^ ± 17.14 ^a^	1.44 × 10^2^ ± 0.66 ^ab^
6-Methyl-5-hepten-2-one	C110930	C_8_H_14_O	2.58 × 10^2^ ± 12.64 ^b^	2.71 × 10^2^ ± 20.57 ^b^	2.60 × 10^2^ ± 4.46 ^b^	3.16 × 10^3^ ± 18.04 ^a^

Note: The data are presented as average values ± SD (n = 3). Averages featuring varying lowercase letters within the same row show a notable difference (*p* < 0.05). YC: *L. bulgaricus* G119: *S. thermophilus* Q019 (1:1); Y1: YC + 3% *W. cibaria* G232; Y2: YC + 5% *W. cibaria* G232; Y3: YC + 7% *W. cibaria* G232.

## Data Availability

The original contributions presented in the study are included in the article, further inquiries can be directed to the corresponding authors.
